# ncRNAs-mediated TIMELESS overexpression in lung adenocarcinoma correlates with reduced tumor immune cell infiltration and poor prognosis

**DOI:** 10.1371/journal.pone.0296829

**Published:** 2024-01-23

**Authors:** Xinliang Gao, Mingbo Tang, Suyan Tian, Jialin Li, Shixiong Wei, Shucheng Hua, Wei Liu

**Affiliations:** 1 Department of Thoracic Surgery, The First Hospital of Jilin University, Changchun, Jilin Province, PR China; 2 Division of Clinical Research, The First Hospital of Jilin University, Changchun, Jilin Province, PR China; 3 Department of Respiratory Medicine, Center for Pathogen Biology and Infectious Diseases, Changchun, Jilin Province, PR China; Xiangya Hospital Central South University, CHINA

## Abstract

Lung adenocarcinoma (LUAD) has a poor prognosis. Circadian genes such as *TIMELESS* have been associated with several pathologies, including cancer. The expression of *TIMELESS* and the relationship between *TIMELESS*, infiltration of tumors and prognosis in LUAD requires further investigation. In this study, we investigated the expression of *TIMELESS* and its association with survival across several types of human cancer using data from The Cancer Genome Atlas (TCGA) and the Genotype-Tissue Expression Program. Noncoding RNAs (ncRNAs) regulating overexpression of *TIMELESS* in lung adenocarcinoma (LUAD) were explored with expression, correlation, and survival analyses. Immune cell infiltration and biomarkers were analyzed between different *TIMELESS* expression levels. The relationship between *TIMELESS* expression and immunophenoscores, which were used to predict response to immunotherapy, was evaluated. *TIMELESS* was identified as a potential oncogene in LUAD. NcRNA analysis showed MIR4435-2HG/hsa-miR-1-3p may interact with *TIMELESS* in a competitive endogenous RNA network in LUAD tumor tissues. Most immune cells were significantly decreased in TCGA LUAD tumor tissues with high *TIMELESS* expression except for CD4^+^T cells and Th2 cells. *TIMELESS* expression in LUAD tumor tissues was significantly negatively correlated with neutrophil biomarkers, dendritic cell biomarkers (*HLA-DPB1*, *HLA-DQB1*, *HLA-DRA*, *HLA-DPA1*, *CD1C*) and an immunophenoscore that predicted outcomes associated with the use of immune checkpoint inhibitors. These findings imply that ncRNAs-mediated *TIMELESS* overexpression in LUAD tumor tissues correlated with poor prognosis, reduced immune cell infiltration in the tumor microenvironment, and poor response to immune checkpoint inhibitors.

## Introduction

In 2020, lung cancer was responsible for approximately 1.8 million deaths, and was the most common cause of cancer-related mortality [1]. Non-small cell lung cancer (NSCLC) is a key type of lung cancer. Lung adenocarcinoma (LUAD) is the most common subtype of NSCLC, and incidence rates have continued to increase in recent years [2]. The application of novel diagnostic and therapeutic technologies, including immunotherapy [3, 4], has improved prognosis in NSCLC, but the overall five-year survival rate remains low, at 25% according to The Surveillance, Epidemiology, and End Results (SEER) [5]. There is clinical need for predictive biomarkers and novel therapeutic strategies in LUAD.

The molecular circadian clock is an endogenous timing system that has been associated with several pathologies, including cancer [6, 7]. *TIMELESS* is a core circadian gene that controls circadian rhythmicity. *TIMELESS* may be involved in carcinogenesis by modulating cell cycles, DNA repair, and tumor immunity [8–13]. The expression of *TIMELESS* is increased and associated with poor patient outcomes in NSCLC [14, 15]. The expression of *TIMELESS* and the association of *TIMELESS* with tumor immune cell infiltration and prognosis in LUAD remain to be elucidated.

Competitive endogenous RNAs (ceRNAs), including long non-coding RNAs (lncRNA), pseudogene transcripts, and circular RNAs (circRNAs), regulate each other as they share microRNA (miRNA) recognition elements [16]. lncRNAs regulate transcription in mammalian circadian systems [17, 18]. The objectives of this study were to investigate: 1) the expression of *TIMELESS* and its association with survival across several types of human cancer; 2) the regulation of *TIMELESS* by non-coding RNAs (ncRNAs) in LUAD; and 3) the relationship between *TIMELESS* and infiltration of tumors in LUAD to predict outcomes associated with the use of immune checkpoint inhibitors. Findings indicate that ncRNA mediate upregulation of *TIMELESS*, which is associated with tumor immune cell infiltration and poor prognosis in LUAD.

## Materials and methods

### Differential genes expression

In the 33 tumor classes included in The Cancer Genome Atlas (TCGA) datasets, we selected 18 tumor types that contained more than five normal tissue samples (Bladder Urothelial Carcinoma BLCA, Breast Invasive Carcinoma BRCA, Cholangiocarcinoma CHOL, Colon Adenocarcinoma COAD, Esophageal Carcinoma ESCA, Glioblastoma Multiforme GBM, Head and Neck Squamous Cell Carcinoma HNSC, Kidney Chromophobe KICH, Kidney Renal Clear Cell Carcinoma KIRC, Kidney Renal Papillary Cell Carcinoma KIRP, Liver Hepatocellular Carcinoma LIHC, Lung Adenocarcinoma LUAD, Lung Squamous Cell Carcinoma LUSC, Prostate Adenocarcinoma PRAD, Rectum adenocarcinoma READ, Stomach Adenocarcinoma STAD, Thyroid Carcinoma THCA, and Uterine Corpus Endometrial Carcinoma UCEC). The mRNA expression data from these 18 cancers were downloaded from TCGA database. Differential expression analysis using the Limma package in R (version 4.0.1) identified altered *TIMELESS* expression between tumors and adjacent normal tissues. The Gene Expression Profiling Interactive Analysis database (GEPIA) (http://gepia.cancer-pku.cn/) [19] was used to compare and visualize TCGA tumor tissues to normal tissues from TCGA and individuals without cancer from the Genotype Tissue Expression (GTEx) Program.

### GEPIA database analysis

Package survival in R was used to determine the overall survival (OS) and disease-free survival (RFS) between different *TIMELESS* expression and lncRNA expression across multiple cancer types, including LUAD. Two groups of high and low expression were established using the median value as a cut-off.

### Candidate miRNA and lncRNA prediction

StarBase (sRNA target Base) (http://starbase.sysu.edu.cn/) [20] was used to identify miRNA binding sites in *TIMELESS* and lncRNAs that could bind candidate miRNAs involved in the regulation of *TIMELESS*. miRNA binding sites were predicted by the following programs: PITA, RNA22, miRmap, microT, miRanda, PicTar, and TargetScan. Candidate miRNAs and lncRNAs were identified based on correlations of miRNA expression (spearman |R| >0.2 p<0.05) or lncRNA expression (spearman |R| >0.1 p<0.05) with *TIMELESS* expression (analyzed and visualized by function cor.test and package ggplot2 in R).

### Tumor immune cell infiltration and response to immune checkpoint inhibitors

Single-sample gene set enrichment analysis (ssGSEA) was used to investigate the proportions of 28 types of immune cells in immune cell infiltration in TCGA LUAD tumor tissues [21]. The Cancer Immunome Atlas (TCIA) (https://tcia.at/) was used to predict response to immune checkpoint inhibitors using an immunophenoscore that predicts response to immunotherapy with Cytotoxic T Lymphocyte-Associated Antigen-4 (CTLA-4) and Programmed Death 1(PD-1) blockers [22]. Immunophenoscores were correlated with *TIMELESS* expression using Spearman’s correlation.

### Statistical analysis

Statistical analyses were performed using online databases or R software, as described above. p <0.05 was considered statistically significant.

## Results

### *TIMELESS* expression and prognostic utility

**Fig 1:**
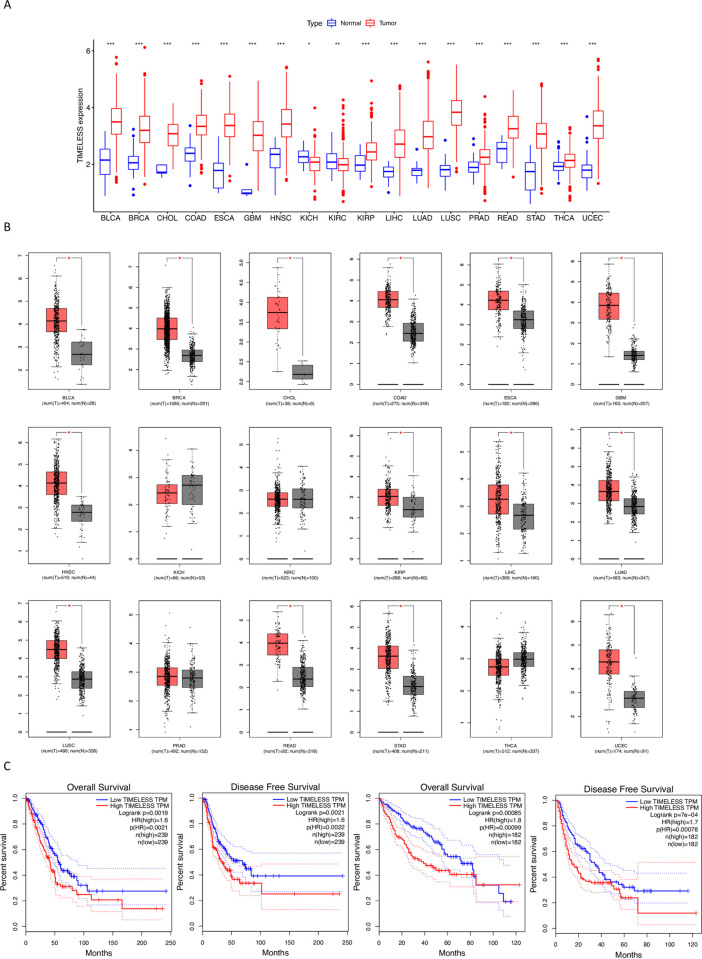
*TIMELESS* expression and prognostic value across multiple types of cancer. (A) *TIMELESS* expression in tumor tissues compared to adjacent normal tissues across 18 types of cancer in TCGA. (B) *TIMELESS* expression in TCGA tumor tissues (red) across18 types of cancer compared to normal tissues (grey) from TCGA and individuals without cancer from the GTEx Program. (C) The prognostic value of *TIMELESS* in LUAD (right two figures) and LIHC (left two figures). *p value < 0.05; **p value < 0.01; ***p value < 0.001.

Data from TCGA showed *TIMELESS* expression in tumor tissues was significantly increased vs. adjacent normal tissues in 16 types of cancer, and significantly decreased vs. adjacent normal tissues in 2 types of cancer (**Fig 1A**). *TIMELESS* expression was significantly increased in TCGA tumor tissues across 14 types of cancer vs. normal tissues from individuals without cancer in the GTEx Program (**Fig 1B**). Among these 14 tumor tissues, high *TIMELESS* expression in tumor tissues was significantly associated with poor OS in KIRC, LIHC, and LUAD and poor RFS in LIHC and LUAD (**Fig 1C**). These data suggest *TIMELESS* may be utilized as a prognostic marker in LUAD.

### *TIMELESS* upstream miRNAs

**Fig 2:**
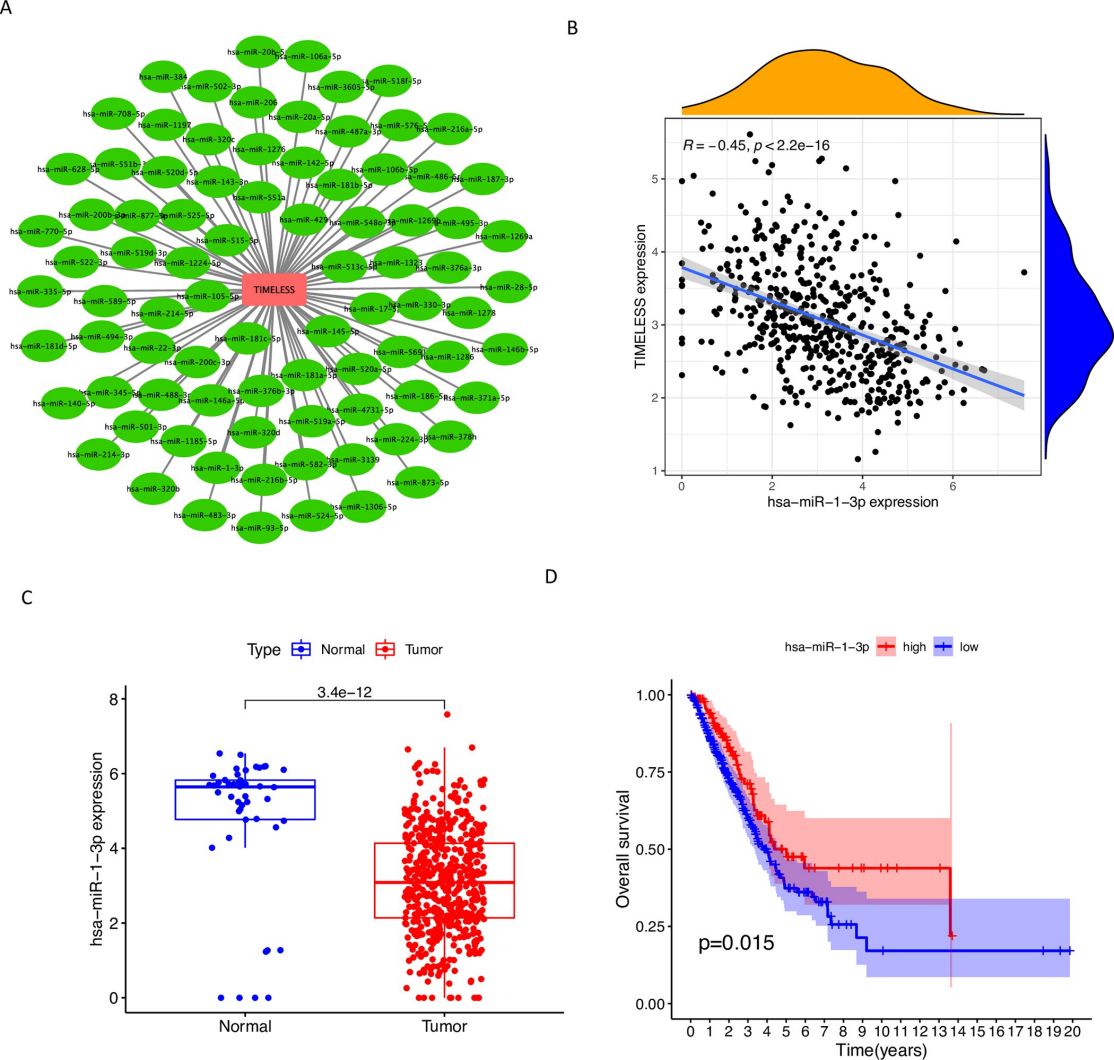
Identification of hsa-miR-1-3p as a potential upstream miRNA of *TIMELESS* in LUAD (A) The miRNA-*TIMELESS* interaction network (Cytoscape software). (B) Correlation between *TIMELESS* expression and expression of candidate miRNAs in TCGA LUAD tumor tissues (starBase database). (C) hsa-miR-1-3p expression in TCGA LUAD tumor tissues compared to adjacent normal tissues. (D) The prognostic value of hsa-miR-1-3p in LUAD.

A total of 85 miRNAs with potential to interact with *TIMELESS* were identified in TCGA LUAD tumor tissues (**Fig 2A**). According to published literature, there is negative feedback regulation of the target gene by miRNAs; therefore, we expected a negative correlation between candidate miRNA and *TIMELESS* expression and downregulation of candidate miRNAs in LUAD tumor tissues vs. adjacent normal tissues. In TCGA LUAD tumor tissues, *TIMELESS* expression showed a significant negative correlation with hsa-miR-1-3p, hsa-miR-145-5p, hsa-miR-181a-5p, hsa-miR-224-3p and hsa-miR-551a expression (**Table 1; Fig 2B**). Only hsa-miR-1-3p was significantly downregulated in LUAD tumor tissues vs. adjacent normal tissues (**Fig 2C**). In the TCGA LUAD cohort, high hsa-miR-1-3p expression in tumor tissues was significantly associated with better OS (**Fig 2D**). These data imply that hsa-miR-1-3p may regulate *TIMELESS* in LUAD.

**Table 1:** Correlations between miRNA and *TIMELESS* expression in TCGA LUAD tumor tissues.

miRNA	R value	P value	logFC	P value
hsa-miR-1-3p	-0.452	<0.001	-1.685	<0.001
hsa-miR-145-5p	-0.265	<0.001	-0.492	0.308
hsa-miR-181a-5p	-0.256	<0.001	-0.099	0.282
hsa-miR-551a	-0.224	<0.001	0.505	<0.001
hsa-miR-224-3p	-0.202	<0.001	0.660	<0.001

### hsa-miR-1-3p upstream lncRNAs

**Fig 3:**
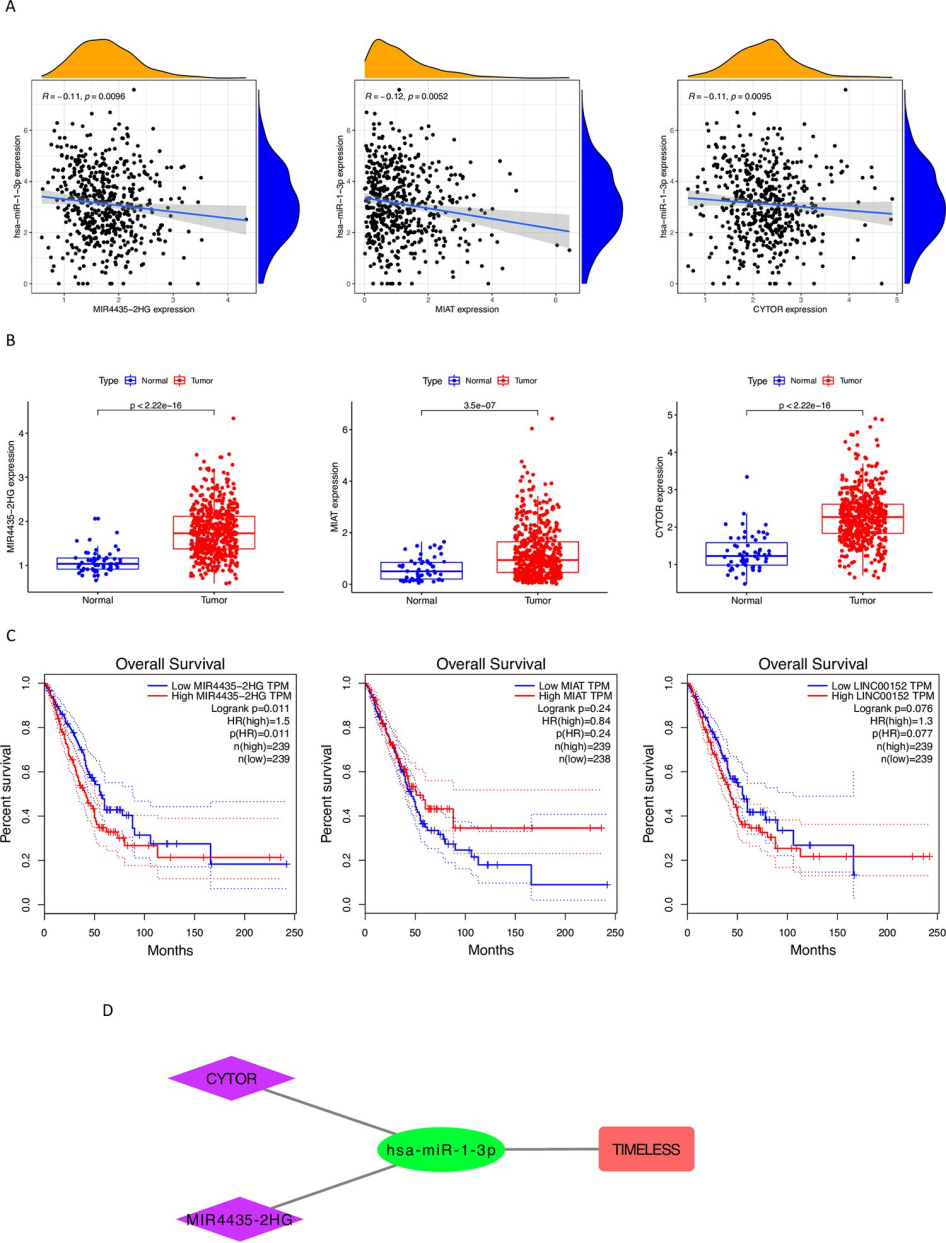
Identification of potential upstream lncRNAs of hsa-miR-1-3p in LUAD. (A) Correlation between hsa-miR-1-3p expression and the expression of candidate lncRNAs (MIR4435−2HG, MIAT, and CYTOR) in TCGA LUAD tumor tissues. (B) Candidate lncRNA (MIR4435−2HG, MIAT, and CYTOR) expression in TCGA LUAD tumor tissues compared to adjacent normal tissues. (C) The prognostic value of candidate lncRNAs (MIR4435−2HG, MIAT, and CYTOR) in LUAD. (D) The lncRNA—hsa-miR-1-3p - *TIMELESS* interaction network in LUAD tumor tissue.

A total of 92 lncRNAs with potential to interact with hsa-miR-1-3p were identified in TCGA LUAD tumor tissues. The lncRNAs most likely to participate in the lncRNA—hsa-miR-1-3p - *TIMELESS* interaction network in LUAD tumor tissue were selected according to the following criteria: significant upregulation of lncRNA expression in LUAD tumor tissues vs. adjacent normal tissues, significant negative correlation between lncRNA expression and hsa-miR-1-3p expression in LUAD tumor tissues, and significant association of high lncRNA expression in tumor tissues with poor prognosis in LUAD. Among the 92 lncRNAs with potential to interact with hsa-miR-1-3p, only *MIR4435-2HG*, *MIAT*, and *CYTOR (LINC00152)* were significantly upregulated in TCGA LUAD tumor tissues vs. adjacent normal tissues, with expression that significantly negatively correlated with hsa-miR-1-3p expression (**Fig 3A and 3B**). High *MIR4435-2HG* expression in tumor tissues was significantly associated with poor OS in the TCGA LUAD cohort (**Fig 3C**). Overexpressed *CYTOR* tended towards poor OS in LUAD but did not reach significance. These data suggest *MIR4435-2HG* and *CYTOR* have a potential role in the lncRNA—hsa-miR-1-3p - *TIMELESS* interaction network in LUAD (**Fig 3D**).

### Tumor immune cell infiltration and response to immune checkpoint inhibitors

**Fig 4:**
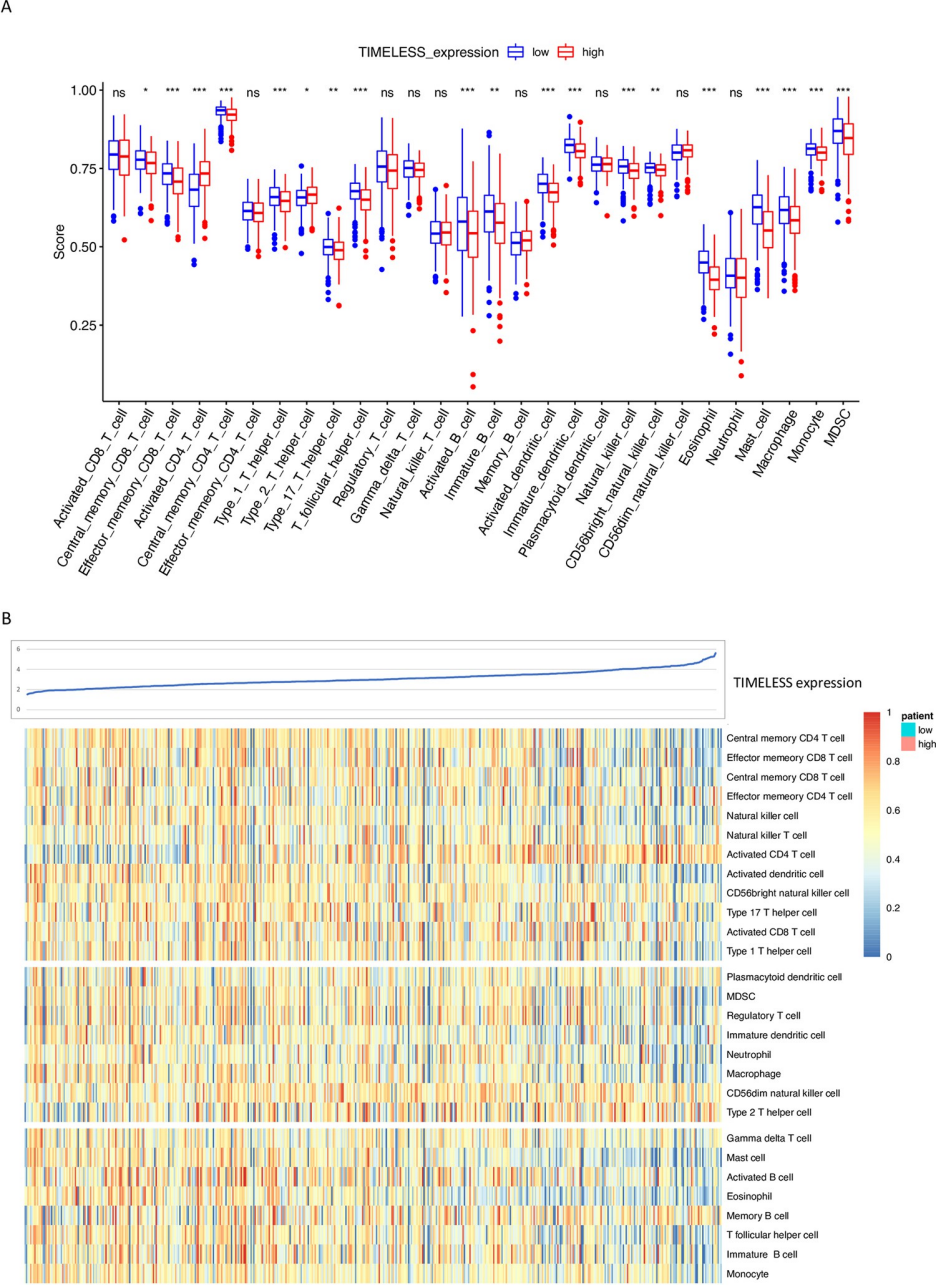
Tumor immune cell infiltration and *TIMELESS* expression in LUAD. (A) Correlation between *TIMELESS* expression and immune cell infiltration in TCGA LUAD tumor tissues. (B) Heatmap of immune cell infiltration in TCGA LUAD tumor tissues stratified by *TIMELESS* expression level. *p value < 0.05; **p value < 0.01; ***p value < 0.001.

**Fig 5:**
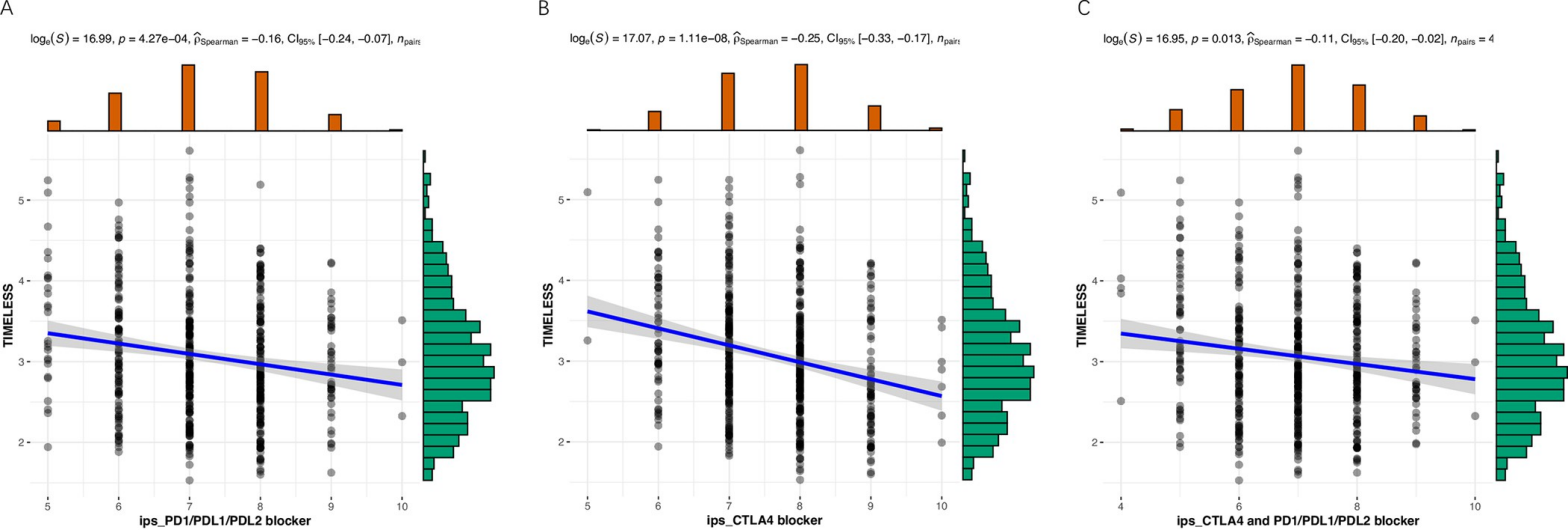
Correlation of *TIMELESS* expression with immunophenoscores that predict response to immune checkpoint inhibitors in TCGA LUAD tumor tissues.

*TIMELESS* plays a critical role in the immune system. ssGSEA scores for most immune cells were significantly decreased in TCGA LUAD tumor tissues with high *TIMELESS* expression vs. low *TIMELESS* expression. ssGSEA scores for activated CD4^+^T cells and the immunosuppressive Th2 cells were significantly increased in TCGA LUAD tumor tissues with high *TIMELESS* expression vs. low *TIMELESS* expression (**Fig 4A**). A heatmap of immune cell infiltration confirmed that increased *TIMELESS* expression was associated with CD4^+^ T and Th2 cell infiltration in TCGA LUAD tumor tissues (**Fig 4B**). *TIMELESS* expression was significantly negatively correlated with neutrophil biomarkers (*CEACAM8*) and dendritic cell biomarkers (*HLA-DPB1*, *HLA-DQB1*, *HLA-DRA*, *HLA-DPA1*, *CD1C*) in TCGA LUAD tumor tissues (**Table 2**). These data suggest *TIMELESS* is involved in immune regulation of the LUAD tumor microenvironment. *TIMELESS* expression was significantly negatively correlated with an immunophenoscore that predicted response to immune checkpoint inhibitors in TCGA LUAD tumor tissues (**Fig 5**). These data suggest patients with LUAD and high *TIMELESS* expression in their tumor tissue may be less responsive to immune checkpoint inhibitors than patients with lower *TIMELESS* expression in their tumor tissue.

**Table 2:** Correlations between *TIMELESS* expression and biomarkers of immune cells in TCGA LUAD tumor tissues.

Immune Cell	Gene	Spearman R	P value
B cell	CD19	0.011	0.804
	CD79A	-0.022	0.612
CD8+ T cell	CD8A	0.080	0.066
	CD8B	0.112	0.010
CD4+ T cell	CD4	-0.188	<0.001
M1 macrophage	NOS2	0.075	0.086
	IRF5	-0.006	0.888
	PTGS2	0.039	0.372
M2 macrophage	CD163	0.002	0.959
	VSIG4	-0.103	0.018
	MS4A4A	-0.172	<0.001
Neutrophil	CEACAM8	-0.325	<0.001
	ITGAM	-0.161	<0.001
	CCR7	-0.178	<0.001
Dendritic cell	HLA-DPB1	-0.385	<0.001
	HLA-DQB1	-0.283	<0.001
	HLA-DRA	-0.353	<0.001
	HLA-DPA1	-0.316	<0.001
	CD1C	-0.531	<0.001
	NRP1	-0.166	<0.001
	ITGAX	-0.020	0.639

## Discussion

This study used data from TCGA and the GTEx Program to explore *TIMELESS* expression and survival outcomes in human cancers. High *TIMELESS* expression in tumor tissue was significantly associated with poor OS in KIRC, LIHC, and LUAD and poor RFS in LIHC and LUAD. Consistent with this, other studies have shown a correlation between *TIMELESS* overexpression and poor prognosis in lung cancer [14, 15, 23], and *TIMELESS* has been used to build a predictive model of survival in lung cancer [24]. However, the regulation and mechanism of action of *TIMELESS* in lung cancer remains to be elucidated.

ncRNAs, including miRNAs and lncRNAs, are one component of post transcriptional regulation of gene expression. In the present study, *TIMELESS* expression was significantly negatively correlated with hsa-miR-1-3p expression in LUAD tumor tissues, and high hsa-miR-1-3p expression was significantly associated with improved OS in LUAD. Previous reports indicate that miR-1-3p has an inhibitory role in lung cancer by targeting *CELSR3* and *FAM83A* and modulating the viability, migration, and invasion of tumor cells [25–27]. According to the ceRNA hypothesis [28], lncRNAs in the lncRNA—hsa-miR-1-3p - *TIMELESS* interaction network in LUAD should be oncogenic. Expression, survival and correlation analyses identified *MIR4435−2HG* as a candidate oncogenic lncRNA in the lncRNA—hsa-miR-1-3p - *TIMELESS* interaction network in LUAD. Previous studies show lnc *MIR4435-2HG* may act as an oncogene across several tumor types; specifically, *MIR4435−2HG* may promote lung cancer by activating TGF-β1 and β-catenin signaling [29–32].

Research has revealed that some genes including circadian genes can alter tumor immune cell infiltration and influence prognosis [12, 33–35]. The present study adds to this evidence-base. High *TIMELESS* expression was associated with decreased infiltration of most immune cells in LUAD tumor tissues. Notably, high *TIMELESS* expression was associated with increased infiltration of activated CD4^+^ T cells and immunosuppressive Th2 cells and decreased infiltration of CD8^+^ T cells, cytotoxic Th1 cells, and dendritic cells in LUAD tumor tissues. These data imply *TIMELESS* is involved in immune regulation of the LUAD tumor microenvironment. Accordingly, a high percent of CD4^+^ T cells in the tumor microenvironment may predict poor prognosis in NSCLC [36], and cytotoxic Th1 cells and dendritic cells can maintain anti-tumor immunity [36]. Further, *TIMELESS* expression in LUAD tumor tissues was significantly negatively correlated with an immunophenoscore that predicted response to immune checkpoint inhibitors [22], reflecting the impact of *TIMELESS* on immune cell infiltration. As is a circadian gene, immune checkpoint inhibitor regimens may need to be optimally timed with a patient’s intrinsic rhythms to improve outcomes. Currently, only chronoradiotherapy and chronochemotherapy have been investigated across several cancers [37–39].

This study had some limitations. First, data were extracted from publicly available databases. Second, immunity-related analyses were based on bioinformatics, with no physiological validation. Third, lung cancer is associated with multiple molecular abnormalities, but we only focused on the lncRNA—miRNA—interaction network, potentially excluding other critical genes.

## Conclusions

In summary, this study identified *TIMELESS* as a potential oncogene across multiple cancers, including LUAD. High *TIMELESS* expression in tumor tissues was significantly associated with poor OS and RFS in LUAD. *MIR4435-2HG* and hsa-miR-1-3p may interact with *TIMELESS* in a ceRNA network in LUAD tumor tissues. *TIMELESS* might exert its oncogenic role by decreasing immune cell infiltration in the LUAD tumor microenvironment, and *TIMELESS* expression in LUAD tumor tissues may predict a poor response to immune checkpoint inhibitors. Basic science and clinical trials are required to validate these findings.

## References

[1] Sung H, Ferlay J, Siegel RL, Laversanne M, Soerjomataram I, Jemal A, et al. Global Cancer Statistics 2020: GLOBOCAN Estimates of Incidence and Mortality Worldwide for 36 Cancers in 185 Countries. CA Cancer J Clin. 2021;71(3):209–49. Epub 2021/02/05. doi: 10.3322/caac.21660 . 33538338

[2] Cheng TY, Cramb SM, Baade PD, Youlden DR, Nwogu C, Reid ME. The International Epidemiology of Lung Cancer: Latest Trends, Disparities, and Tumor Characteristics. Journal of thoracic oncology: official publication of the International Association for the Study of Lung Cancer. 2016;11(10):1653–71. Epub 2016/07/02. doi: 10.1016/j.jtho.2016.05.021 ; PubMed Central PMCID: PMC5512876. 27364315 PMC5512876

[3] Peters S, Reck M, Smit EF, Mok T, Hellmann MD. How to make the best use of immunotherapy as first-line treatment of advanced/metastatic non-small-cell lung cancer. Annals of oncology: official journal of the European Society for Medical Oncology. 2019;30(6):884–96. Epub 2019/03/27. doi: 10.1093/annonc/mdz109 . 30912805

[4] Hirsch FR, Suda K, Wiens J, Bunn PA Jr., New and emerging targeted treatments in advanced non-small-cell lung cancer. Lancet (London, England). 2016;388(10048):1012–24. Epub 2016/09/07. doi: 10.1016/s0140-6736(16)31473-8 . 27598681

[5] Siegel RL, Miller KD, Jemal A. Cancer statistics, 2019. CA: a cancer journal for clinicians. 2019;69(1):7–34. Epub 2019/01/09. doi: 10.3322/caac.21551 . 30620402

[6] Xuan W, Khan F, James CD, Heimberger AB, Lesniak MS, Chen P. Circadian regulation of cancer cell and tumor microenvironment crosstalk. Trends Cell Biol. 2021. Epub 2021/07/18. doi: 10.1016/j.tcb.2021.06.008 . 34272133 PMC8526375

[7] Pariollaud M, Lamia KA. Cancer in the Fourth Dimension: What Is the Impact of Circadian Disruption? Cancer Discov. 2020;10(10):1455–64. Epub 2020/09/17. doi: 10.1158/2159-8290.CD-20-0413 ; PubMed Central PMCID: PMC7541588. 32934020 PMC7541588

[8] Li B, Mu L, Li Y, Xia K, Yang Y, Aman S, et al. TIMELESS inhibits breast cancer cell invasion and metastasis by down-regulating the expression of MMP9. Cancer Cell Int. 2021;21(1):38. Epub 2021/01/13. doi: 10.1186/s12935-021-01752-y ; PubMed Central PMCID: PMC7798230. 33430865 PMC7798230

[9] Cao M, Wang Y, Xiao Y, Zheng D, Zhi C, Xia X, et al. Activation of the clock gene TIMELESS by H3k27 acetylation promotes colorectal cancer tumorigenesis by binding to Myosin-9. J Exp Clin Cancer Res. 2021;40(1):162. Epub 2021/05/12. doi: 10.1186/s13046-021-01936-4 ; PubMed Central PMCID: PMC8108341. 33971927 PMC8108341

[10] Kang TH, Reardon JT, Sancar A. Regulation of nucleotide excision repair activity by transcriptional and post-transcriptional control of the XPA protein. Nucleic Acids Res. 2011;39(8):3176–87. Epub 2011/01/05. doi: 10.1093/nar/gkq1318 ; PubMed Central PMCID: PMC3082913. 21193487 PMC3082913

[11] Unsal-Kaçmaz K, Mullen TE, Kaufmann WK, Sancar A. Coupling of human circadian and cell cycles by the timeless protein. Mol Cell Biol. 2005;25(8):3109–16. Epub 2005/03/31. doi: 10.1128/MCB.25.8.3109-3116.2005 ; PubMed Central PMCID: PMC1069621. 15798197 PMC1069621

[12] Wang Z, Su G, Dai Z, Meng M, Zhang H, Fan F, et al. Circadian clock genes promote glioma progression by affecting tumour immune infiltration and tumour cell proliferation. Cell Prolif. 2021;54(3):e12988. Epub 2021/01/15. doi: 10.1111/cpr.12988 ; PubMed Central PMCID: PMC7941241. 33442944 PMC7941241

[13] Xing X, Gu F, Hua L, Cui X, Li D, Wu Z, et al. TIMELESS Promotes Tumor Progression by Enhancing Macrophages Recruitment in Ovarian Cancer. Front Oncol. 2021;11:732058. Epub 2021/09/08. doi: 10.3389/fonc.2021.732058 ; PubMed Central PMCID: PMC8417241. 34490127 PMC8417241

[14] Zhang Y, Peng X, Yang H, Zhao H, Xia B, You Y. The expression of the circadian gene TIMELESS in non-small-cell lung cancer and its clinical significance. Int J Clin Exp Pathol. 2020;13(9):2297–304. Epub 2020/10/13. ; PubMed Central PMCID: PMC7539872. 33042334 PMC7539872

[15] Yoshida K, Sato M, Hase T, Elshazley M, Yamashita R, Usami N, et al. TIMELESS is overexpressed in lung cancer and its expression correlates with poor patient survival. Cancer Sci. 2013;104(2):171–7. Epub 2012/11/24. doi: 10.1111/cas.12068 ; PubMed Central PMCID: PMC4454395. 23173913 PMC4454395

[16] Thomson DW, Dinger ME. Endogenous microRNA sponges: evidence and controversy. Nat Rev Genet. 2016;17(5):272–83. Epub 2016/04/05. doi: 10.1038/nrg.2016.20 . 27040487

[17] Li H, Xu LX, Yu J, Tan L, Miao P, Yang X, et al. The role of a lncRNA (TCONS_00044595) in regulating pineal CLOCK expression after neonatal hypoxia-ischemia brain injury. Biochem Biophys Res Commun. 2020;528(1):1–6. Epub 2020/05/26. doi: 10.1016/j.bbrc.2020.05.047 . 32448507

[18] Fan Z, Zhao M, Joshi PD, Li P, Zhang Y, Guo W, et al. A class of circadian long non-coding RNAs mark enhancers modulating long-range circadian gene regulation. Nucleic Acids Res. 2017;45(10):5720–38. Epub 2017/03/24. doi: 10.1093/nar/gkx156 ; PubMed Central PMCID: PMC5449593. 28335007 PMC5449593

[19] Li C, Tang Z, Zhang W, Ye Z, Liu F. GEPIA2021: integrating multiple deconvolution-based analysis into GEPIA. Nucleic Acids Res. 2021;49(W1):W242–w6. Epub 2021/05/30. doi: 10.1093/nar/gkab418 ; PubMed Central PMCID: PMC8262695. 34050758 PMC8262695

[20] Li JH, Liu S, Zhou H, Qu LH, Yang JH. starBase v2.0: decoding miRNA-ceRNA, miRNA-ncRNA and protein-RNA interaction networks from large-scale CLIP-Seq data. Nucleic Acids Res. 2014;42(Database issue):D92–7. Epub 2013/12/04. doi: 10.1093/nar/gkt1248 ; PubMed Central PMCID: PMC3964941. 24297251 PMC3964941

[21] Bindea G, Mlecnik B, Tosolini M, Kirilovsky A, Waldner M, Obenauf AC, et al. Spatiotemporal dynamics of intratumoral immune cells reveal the immune landscape in human cancer. Immunity. 2013;39(4):782–95. Epub 2013/10/22. doi: 10.1016/j.immuni.2013.10.003 . 24138885

[22] Charoentong P, Finotello F, Angelova M, Mayer C, Efremova M, Rieder D, et al. Pan-cancer Immunogenomic Analyses Reveal Genotype-Immunophenotype Relationships and Predictors of Response to Checkpoint Blockade. Cell Rep. 2017;18(1):248–62. Epub 2017/01/05. doi: 10.1016/j.celrep.2016.12.019 . 28052254

[23] Qiu M, Chen YB, Jin S, Fang XF, He XX, Xiong ZF, et al. Research on circadian clock genes in non-small-cell lung carcinoma. Chronobiol Int. 2019;36(6):739–50. Epub 2019/04/25. doi: 10.1080/07420528.2018.1509080 . 31014124

[24] Gao X, Tang M, Tian S, Li J, Liu W. Identification of a circadian gene signature that predicts overall survival in lung adenocarcinoma. PeerJ. 2021;9:e11733. Epub 2021/07/22. doi: 10.7717/peerj.11733 ; PubMed Central PMCID: PMC8272922. 34285836 PMC8272922

[25] Cheng R, Zhang G, Bai Y, Zhang F, Zhang G. LncRNA SENCR promotes cell proliferation and progression in non-small-cell lung cancer cells via sponging miR-1-3p. Cell Cycle. 2021;20(14):1402–14. Epub 2021/07/06. doi: 10.1080/15384101.2021.1924958 ; PubMed Central PMCID: PMC8344740. 34224326 PMC8344740

[26] Miao H, Zeng Q, Xu S, Chen Z. miR-1-3p/CELSR3 Participates in Regulating Malignant Phenotypes of Lung Adenocarcinoma Cells. Curr Gene Ther. 2021;21(4):304–12. Epub 2021/06/19. doi: 10.2174/1566523221666210617160611 . 34139980

[27] Liu PJ, Chen YH, Tsai KW, Yeah HY, Yeh CY, Tu YT, et al. Involvement of MicroRNA-1-FAM83A Axis Dysfunction in the Growth and Motility of Lung Cancer Cells. Int J Mol Sci. 2020;21(22). Epub 2020/12/04. doi: 10.3390/ijms21228833 ; PubMed Central PMCID: PMC7700477. 33266425 PMC7700477

[28] Salmena L, Poliseno L, Tay Y, Kats L, Pandolfi PP. A ceRNA hypothesis: the Rosetta Stone of a hidden RNA language? Cell. 2011;146(3):353–8. Epub 2011/08/02. doi: 10.1016/j.cell.2011.07.014 ; PubMed Central PMCID: PMC3235919. 21802130 PMC3235919

[29] Shen W, Pu J, Sun J, Tan B, Wang W, Wang L, et al. Zebrafish xenograft model of human lung cancer for studying the function of LINC00152 in cell proliferation and invasion. Cancer Cell Int. 2020;20:376. Epub 2020/08/11. doi: 10.1186/s12935-020-01460-z ; PubMed Central PMCID: PMC7409423. 32774169 PMC7409423

[30] Zhang J, Li W. Long noncoding RNA CYTOR sponges miR-195 to modulate proliferation, migration, invasion and radiosensitivity in nonsmall cell lung cancer cells. Biosci Rep. 2018;38(6). Epub 2018/11/30. doi: 10.1042/BSR20181599 ; PubMed Central PMCID: PMC6435535. 30487160 PMC6435535

[31] Yang M, He X, Huang X, Wang J, He Y, Wei L. LncRNA MIR4435-2HG-mediated upregulation of TGF-β1 promotes migration and proliferation of nonsmall cell lung cancer cells. Environ Toxicol. 2020;35(5):582–90. Epub 2019/12/26. doi: 10.1002/tox.22893 . 31875359

[32] Qian H, Chen L, Huang J, Wang X, Ma S, Cui F, et al. The lncRNA MIR4435-2HG promotes lung cancer progression by activating β-catenin signalling. J Mol Med (Berl). 2018;96(8):753–64. Epub 2018/06/07. doi: 10.1007/s00109-018-1654-5 . 29872866

[33] Bai R, Zhang J, He F, Li Y, Dai P, Huang Z, et al. GPR87 promotes tumor cell invasion and mediates the immunogenomic landscape of lung adenocarcinoma. Commun Biol. 2022;5(1):663. Epub 2022/07/06. doi: 10.1038/s42003-022-03506-6 ; PubMed Central PMCID: PMC9256611. 35790819 PMC9256611

[34] Yu Y, Wang Z, Zheng Q, Li J. GREB1L overexpression correlates with prognosis and immune cell infiltration in lung adenocarcinoma. Sci Rep. 2021;11(1):13281. Epub 2021/06/26. doi: 10.1038/s41598-021-92695-x ; PubMed Central PMCID: PMC8225624. 34168239 PMC8225624

[35] Qiu A, Xu H, Mao L, Xu B, Fu X, Cheng J, et al. A Novel apaQTL-SNP for the Modification of Non-Small-Cell Lung Cancer Susceptibility across Histological Subtypes. Cancers (Basel). 2022;14(21). Epub 2022/11/12. doi: 10.3390/cancers14215309 ; PubMed Central PMCID: PMC9658938. 36358727 PMC9658938

[36] Giatromanolaki A, Anestopoulos I, Panayiotidis MI, Mitrakas A, Pappa A, Koukourakis MI. Prognostic Relevance of the Relative Presence of CD4, CD8 and CD20 Expressing Tumor Infiltrating Lymphocytes in Operable Non-small Cell Lung Cancer Patients. Anticancer Res. 2021;41(8):3989–95. Epub 2021/07/21. doi: 10.21873/anticanres.15196 . 34281863

[37] Bermúdez-Guzmán L, Blanco-Saborío A, Ramírez-Zamora J, Lovo E. The Time for Chronotherapy in Radiation Oncology. Front Oncol. 2021;11:687672. Epub 2021/05/29. doi: 10.3389/fonc.2021.687672 ; PubMed Central PMCID: PMC8144648. 34046365 PMC8144648

[38] Redondo JA, Bibes R, Vercauteren Drubbel A, Dassy B, Bisteau X, Maury E, et al. PER2 Circadian Oscillation Sensitizes Esophageal Cancer Cells to Chemotherapy. Biology (Basel). 2021;10(4). Epub 2021/04/04. doi: 10.3390/biology10040266 ; PubMed Central PMCID: PMC8065910. 33810377 PMC8065910

[39] Zarogoulidis P, Darwiche K, Kalamaras G, Huang H, Hohenforst-Schmidt W, Zarogoulidis K. Targeted versus chrono-targeted chemotherapy for inhaled chemotherapy in non-small cell lung cancer. Transl Lung Cancer Res. 2013;2(1):E17–22. Epub 2013/02/01. doi: 10.3978/j.issn.2218-6751.2012.12.07 ; PubMed Central PMCID: PMC4367653. 25806211 PMC4367653

